# Ovarian endometrioma increases the embryo aneuploid rate: an analysis of 7092 biopsied blastocysts from fertile monogenetic disease carriers

**DOI:** 10.1186/s12905-023-02406-z

**Published:** 2023-05-09

**Authors:** Niwei Yan, Xi Yuan, Sunxing Huang, Huiying Jie, Jing Wang, Yuan Yuan

**Affiliations:** 1 Reproductive Medicine Center, The First Affiliated Hospital, Sun Yat-Sen University, 1, Zhongshan Road II, Guangzhou, 510080 China; 2 Department of Obstetrics and Gynecology, National University Hospital, 5 Lower Kent Ridge Road, Singapore, 119228 Singapore

**Keywords:** Endometriosis, Endometrioma, Oocyte, Embryo development, Aneuploid, PGT-M

## Abstract

**Background:**

Endometriosis affects many reproductive aged patients with fertility decline and poor outcomes of assisted reproductive treatments, mainly by decreased ovarian reserve and lower fertilization and implantation rates. In recent decade, altered oocyte microenvironments and abnormal spindle organization have been reported to be critical to oocyte chromosomal segregation, organization and aneuploid formation. However, clinical evidences are still limited on whether endometriosis influences oocyte and embryo development. We aimed to figure out the impact of endometrioma on embryo aneuploid formation.

**Method:**

This retrospective cohort study included 1,021 patients (7,092 biopsied embryos) from January 2012 to December 2020. Fertile patients without a history of miscarriage who underwent PGT-M treatment with aneuploid screening were included. Patients with ovarian endometrioma were defined as the study group, while patients without endometriosis were defined as the control group. All demographic, controlled ovarian stimulation treatment and aneuploid screening data were recorded and compared.

**Results:**

The incidence of endometrioma in our study population was 6.5%. There were 7,092 embryos biopsied in total, with 308 embryos in the study group and 6,784 embryos in the control groups. The demographic characteristics were comparable between the two groups except the basal FSH level (6.02 IU/L vs. 5.52 IU/L, p = 0.012). The euploid rate of the study group was significantly lower than that of the control group (52.6% vs. 61.8%, p = 0.012), while the oocyte maturation, fertilization, usable embryo and blastocyst formation rates were comparable. Adjusted for basal FSH level, starting stimulating gonadotropin dosage, total gonadotropin dosage and FSH level on hCG day, euploid rate was still negatively related to endometrioma status.

**Conclusions:**

Endometrioma status disturbs oocyte and embryo development. For infertile patients with endometrioma who require assisted reproductive treatment, pre-treatment is necessary to improve treatment outcomes.

**Trial registration:**

Not applicable.

## Background

Endometriosis is a common chronic inflammatory disease for women of reproductive age, with a prevalence of 6–10% in the general female population [1]. With the progressive disruption of the pelvic and ovarian environment, fertility decline is the main complaint of many patients [2]. However, infertile patients with endometriosis seeking assisted reproductive treatments are also associated with poor clinical outcomes [3–5]. Decreased ovarian reserve and lower fertilization and implantation rates are the main contributors to unpleasant treatment outcomes [6]. In recent years, an increasing number of studies have reported an altered oocyte microenvironment [7] and abnormal spindle organization [8], which are critical to oocyte chromosomal segregation, organization and aneuploid formation. However, real-world clinical data are still limited.

In 2017, Juneau et al. reported that patients with endometriosis undergoing IVF had aneuploidy rates equivalent to their age-matched peers in an IVF population who did not have endometriosis from a sample of 4,103 patients [9]. There were several weaknesses that limited the interpretation of this result. Firstly, the diagnosis of endometriosis was not stratified. Secondly, all the patients enrolled were receiving PGS treatment. There might be other vital confounders of aneuploid involvement that were not properly adjusted during the analysis. Thirdly, all the patients were infertile, which limits the investigation of the pathology of endometriosis itself.

Therefore, we recruited a group of patients with monogenetic disease seeking PGT-M treatment without diagnosis of infertility. The aneuploid rate was calculated according to the endometriosis status. To avoid the heterogeneity of endometriosis, only patients with ovarian endometrioma were enrolled in the study group. With all the above strict settings, we anticipate revealing the bona fide impact of endometrioma on embryo aneuploid formation.

## Methods

This retrospective cohort study was performed from January 2012 to December 2020 in the Reproductive Medicine Center and was approved by the Ethics Committee of The First Affiliated Hospital, Sun Yat-Sen University. Written informed consent was obtained from all the patients for anonymous use of their personal data. All procedures performed in this study involving human participants were in accordance with the Declaration of Helsinki.

### Study population

All patients who underwent PGT-M treatment with aneuploid screening were included. All the clinical files of the patients were scrutinized to verify the diagnosis of endometriosis. Previous history, medical records (surgery) and results of transvaginal ultrasound examination plus histological evidence were the main considerations of endometriosis diagnosis. With the confirmed endometriosis diagnosis, only the aneuploid rate of patients with present ovarian endometrioma was calculated, defined as the study group. Patients without any sign of endometriosis were defined as the control group. Patients with infertility and spontaneous abortion history were excluded from both groups. If the patients had several controlled ovarian stimulation cycles, only the first cycle was analysed. All demographic information, baseline endocrinologic data and controlled ovarian stimulation parameters were recorded.

### Controlled ovarian stimulation and PGT-M

Routine controlled ovarian stimulation protocols were implemented, including an agonist long protocol, an antagonist protocol and mild ovarian stimulation cycles. Both recombined and highly purified urinary gonadotropins were utilized. Final oocyte maturation was typically induced with 6,000 to 10,000 IU of human chorionic gonadotropin when at least three follicles had reached 18 mm in maximal diameter. Transvaginal oocyte retrieval was performed 36 h after human chorionic gonadotropin administration.

Intracytoplasmic sperm injection, embryo culture, blastocyst culture and trophectoderm biopsy were routine procedures. Usable blastocysts were biopsied on Days 5 and 6. Monogenetic disease was diagnosed with specific probes. Aneuploid screening was performed through next-generation sequencing (NGS) or a single-nucleotide polymorphism (SNP) microarray platform. All usable euploid blastocysts were cryopreserved for future use.

### Statistical analysis

Statistical analysis was performed using SPSS version 26 (IBM). The euploid rate was defined as the number of blastocysts biopsied divided by the number of euploids. Continuous data are presented as the mean ± standard deviation, and Student’s t-test was performed for intergroup comparisons. Categorical data are presented as percentages, and the Chi-square test was used for intergroup comparisons. Associations between endometriosis status and euploid rate were assessed using multivariable linear regression. The analysis was adjusted for basal FSH level, starting stimulation gonadotropin dosage, total gonadotropin usage, and FSH level on hCG day. A p value < 0.05 was considered significant. The Post-hoc power analyses were conducted using G*Power (Version 3.1.9.2.).

## Results

In total, 1,021 fertile patients who underwent PGT-M treatment and aneuploid analysis were enrolled in this study. There were 67 patients with confirmed endometriosis diagnosis. The incidence of endometrioma was 6.5%. Fourteen patients were excluded from the final analysis since there was no evidence of ovarian endometrioma according to ultrasonographic examination. Thus, there were 53 patients in the study group and 954 patients in the control group. The rate of bilateral endometriomas in the study group is 60.4% (32/53). The aetiologies of PGT-M treatment were thalassemia, haemophilia, Duchenne’s muscular dystrophy, neurofibroma, spinal muscular atrophy, mucopolysaccharidosis, etc. With a one-sided significance level of 0.05 and our current sample size, the power was 0.815 to detect the difference of euploid rate between the two groups.

The demographic characteristics are summarized in Table 1. Age, BMI, anti-Mullerian hormone level and basal gynaecological endocrinology status were comparable in the two groups except the basal FSH level, which was significantly higher in the study group than in the control group.

**Table 1:** Baseline characteristics of the study population

Parameter	Study group	Control group	P value
Age	31.46 ± 4.14	31.69 ± 4.34	0.699
BMI	21.11 ± 3.18	21.37 ± 2.67	0.493
AMH	3.65 ± 2.74	4.28 ± 3.49	0.280
FSH	6.02 ± 2.05	5.52 ± 1.37	0.012
LH	3.35 ± 1.75	3.57 ± 2.19	0.467
E_2_	34.38 ± 16.44	33.72 ± 18.37	0.801
PRL	18.10 ± 14.49	16.90 ± 14.39	0.558
T	0.28 ± 0.10	0.42 ± 0.20	0.670

Routine controlled ovarian stimulation protocols were applied in our study, such as agonist, antagonist and mild ovarian stimulation protocols. The different protocol proportions in the two groups were equivalent. The starting stimulation dosage in the study group was significantly higher than that in the control group. With a similar stimulation duration, the total gonadotropin usage in the study group was significantly higher than that in the control group. The FSH level on the day of hCG administration was significantly higher than that of the control group. However, the E2 level on the day of hCG administration and the number of oocytes retrieved were comparable between the two groups. Although two different aneuploid screening platforms were used, the proportions in the two groups were comparable. The details are provided in Table 2.

The mature oocyte, fertilization, multipronuclear, cleavage and blastocyst formation rates were similar in the two groups. The usable embryo rate of the study group was lower than that of the control group; however, the difference between the two groups was not statistically significant. There were 7,092 embryos biopsied in total, with 308 embryos in the study group and 6,784 embryos in the control group. The euploid rate was calculated per person but not for the whole group. The euploid rate of the study group was significantly lower than that of the control group. And the mosaicism rate in the study group is 16.32% and 15.41% in the control group. All the details are provided in Table 3.

**Table 2:** Treatment parameters of the study population

Parameter	Study group	Control group	P value
Protocol			0.923
Agonist	66.04% (35)	66.87% (638)	
Antagonist	32.08% (17)	31.87% (304)	
Mild stimulation	1.88% (1)	1.26% (12)	
Starting dosage	244.58 ± 60.02	218.25 ± 59.30	0.002
Total GN	2625.80 ± 916.88	2319.86 ± 843.94	0.011
COS duration	10.45 ± 1.75	10.37 ± 1.81	0.736
FSH on HCG day	16.24 ± 5.78	13.42 ± 5.09	0.000
LH on HCG day	1.15 ± 1.03	1.23 ± 1.35	0.667
E_2_ on HCG day	2696.93 ± 1334.92	2841.02 ± 1155.18	0.430
P on HCG day	1.03 ± 0.48	0.90 ± 0.77	0.218
NO. of oocytes	16.68 ± 8.29	18.33 ± 8.49	0.167
PGT-A Platform			0.535
NGS	75.5% (40)	70.5% (673)	
SNP	24.5% (13)	29.5% (281)	

**Table 3:** Embryonic parameters of the study population

Parameter	Study group	Control group	P value
Mature oocyte	0.828 ± 0.136	0.832 ± 0.133	0.807
Fertilization rate	0.647 ± 0.179	0.670 ± 0.156	0.296
MPN rate	0.022 ± 0.057	0.021 ± 0.055	0.937
Cleavage rate	0.986 ± 0.034	0.989 ± 0.037	0.619
Embryo rate	0.371 ± 0.163	0.414 ± 0.174	0.078
Blastocyst rate	0.685 ± 0.192	0.700 ± 0.192	0.566
Good Blastocyst rate	0.726 ± 0.144	0.704 ± 0.232	0.369
Euploid rate	0.526 ± 0.294	0.618 ± 0.256	0.012

We used multivariable linear regression to analyse the association between endometrioma status and embryonic treatment outcomes and further adjusted for basal FSH level, starting stimulating gonadotropin dosage, total gonadotropin dosage and FSH level on hCG day. Unlike the rest of the factors, the euploid rate was negatively related to endometriosis status. The details are shown in Table 4.

## Discussion

In recent years, with the rapid development of reproductive technology, accumulating evidence has emerged to uncover the relationship between infertility and endometriosis. Although the poor ART treatment outcomes are universally understood [3–5], the specific pathophysiology of endometriosis is still obscure.

**Table 4:** Association between endometrioma status and treatment outcomes

Treatment outcome	Multivariable linear regression		
	**B**	***p***	**95%CI**
Mature oocyte	-0.008	0.704	-0.048—0.032
Fertilization rate	-0.020	0.565	-0.061—0.033
MPN rate	0.005	0.891	-0.016—0.018
Cleavage rate	-0.036	0.302	-0.017—0.005
Embryo rate	-0.045	0.186	-0.087—0.017
Blastocyst rate	-0.008	0.816	-0.065—0.051
Euploid rate	-0.071	0.037	-0.160— -0.005

Note: B for coefficient of independent variable, *p* for significance

Endometriosis is a heterogeneous disease with three well-recognized phenotypes: superficial peritoneal lesions, ovarian endometriomas and deep infiltrating endometriosis [10]. Endometriosis is stratified by the American Society for Reproductive Medicine (ASRM) classification into four stages (I, II, III and IV) according to surgical evaluation of the size, location and severity of endometriotic lesions and the occurrence of extensions of adhesions [11]. However, during everyday practice, not all patients undergo surgery to diagnose endometriosis, which makes it difficult to unify the study population. In our study, based on the history, medical records, ultrasound examination and histological results, we obtained a raw incidence of 6.5%, which was consistent with previous reports [1]. Since our study population was fertile, it makes sense that our incidence of endometriosis was near the lower portion of the previous report.

In 2019, Horton et al. reported the reproductive outcomes of women with endometriosis through a systematic review and meta-analysis. They found that milder forms of endometriosis were most likely to affect the fertilization rate and earlier implantation processes, while the more severe forms of the disease (ASRM III and IV) influenced all stages of reproduction. Ovarian endometriosis negatively affects the oocyte yield and number of mature oocytes [6]. Based on this result, to detect the latent effect of endometriosis on aneuploid formation, which might be minor but does exist, we ruled out the superficial peritoneal lesion phenotype. Ovarian endometrioma confirmed by transvaginal ultrasound examination and histological results was the only criterion of the study group, which means that we covered all the phenotypes that might influence embryo formation and development, even though not all the patients underwent surgery for diagnosis and stratification. Under this strict setting, the mature oocyte, fertilization, cleavage, multiple pronuclear, blastocyst formation rates and usable embryo rate of the endometriosis group were all comparable to those of the control group. The euploid rate of the endometriosis group was significantly lower than the control group. The endometriosis patients in our study group were all fertile, which means that their lesions might be limited to the ovaries without other pelvic tissue infiltration and adhesion. Ovarian space-occupying lesions decreased ovarian reserve [12], however, the difference of serum anti-Mullerian hormone level between the two groups didn’t reach statistically significant level in our study. The oocyte maturation and embryo formation processes were not affected by these ovarian lesions. The mature oocyte, fertilization, cleavage, multiple pronuclear and blastocyst formation rates of the endometriosis group were all comparable with those of the control group. However, the euploid rate in the endometriosis group was significantly lower than that in the control group, which means that the quality of the embryos was also reduced.

Our study results were not consistent with Juneau’s study [9]. A previous study obtained a large sample size of 4,103 patients who underwent PGS treatment. However, their study population was not stratified. For different stages or phenotypes, the pathophysiology of endometriosis is not the same [13–15]. The aneuploid rate might only contribute slightly to the poor treatment outcome. Considering all the phenotypes as a whole for analysis, the chance of detecting minor differences might be missed. In their study, the study population was patients who underwent PGS treatment. Moreover, they did not state the indications of PGS treatment. PGS is utilized in patients of advanced reproductive age, recurrent spontaneous miscarriage or pregnancy loss [16]. The average age of their study population was approximately 36, which was not well accepted as over 38. This means that the majority of their study population was patients with an unpleasant pregnancy history. Age is the most powerful contributor to aneuploidy [17–19]. For age-independent aneuploids, the inherent miosis process was the major influencing factor [20]. If the major study population was patients suffering from recurrent spontaneous miscarriage, it is unsurprising that other minor aetiological factors of aneuploid formation could not be detected. Finally, the study population consisted of infertile patients, meaning that other factors influenced the embryo formation or implantation processes. Similar to their own thoughts, if alterations in the spindle apparatus resulted in developmental arrest before the blastocyst stage, those embryos would not have been included in their analysis. They did not provide much data about embryo development, which makes it hard to interpret their final results. The overall euploid rate in their study was higher than ours, which might be due to the different aneuploid screening platforms [21] since the majority of our study utilized NGS while PCR in their study.

There have been many studies evaluating the impact of the microenvironment of endometriosis patients on oocyte development. In 2009, Barcelos et al. reported no significant differences in the frequency of meiotic anomalies between metaphase II oocytes matured in vitro from MI or GV of infertile patients with endometriosis or not in a preliminary study [22], but with a tendency of more telophase I oocytes in the endometriosis group. In 2013, Dib et al., other researchers from the previous research group, reported that in vivo matured oocytes of infertile patients with endometriosis did not demonstrate significant differences in terms of the nuclear maturation stage, the percentage of oocytes in metaphase II with visible spindles, or spindle localization when compared to the control group under polarization microscopy [23]. However, in 2014, using an animal model, other researchers from the same previous research group reported that bovine oocytes matured in vitro in follicle fluid collected from mild endometriosis patients and had a higher immature rate and percentage of meiotic abnormalities, such as misaligned chromosomes or abnormal spindles [7]. Conflicting data were presented from the same research group as the studies went further, from in vitro to in vivo, from human oocytes to an animal model. Although no conclusion could be drawn about aneuploid formation from their study, they do suggest that the follicular fluid of endometriosis patients may undergo some pathological changes. Therefore, the oocytes that went through in vitro maturation out of this toxic environment might be saved from the error development process, while those still caught in this environment were doomed.

There are many strengths of our study to investigate the impact of endometrioma on aneuploid formation. Firstly, the study population was homogeneous. Although we could not stratify fertile patients with ovarian endometrioma according to the ARSM classification, the number of fertile patients with ovarian endometrioma was equal to that of mild endometriosis patients without extensive pelvic adhesions. Secondly, all the patients were fertile monogenetic disease carriers without an unpleasant pregnancy history. Their inherent oocyte and embryo development processes were relatively normal compared with infertile or recurrent miscarriage patients. Under this setting, the confounding factor of aneuploid formation could be reduced to a minimum. Thirdly, with 7,092 biopsied embryos and 1,021 patients, our study population was large enough to obtain a proper power to detect the difference of euploid rate between the two groups which was tested by the Post-hoc power analyses.

There are still some weaknesses in our study. Firstly, it was a single centre-based retrospective study. Secondly, the study population was monogenetic disease carriers. Evidence concerning embryo development and monogenetic disease is rare. The majority of our study population was patients suffering from thalassemia. Our previous study reported that maternal thalassemia carrier status did not impair ovarian response or embryo development [24]. There are still many other rare monogenetic diseases lacking information or evidence on embryo development.

## Conclusions

Our study found that the aneuploid rate of fertile patients with ovarian endometrioma was increased, although oocyte maturation, fertilization and early development processes were not interfered with. To illustrate the underlying pathological mechanism, in vitro experiments or animal models are needed in the near future.

## Data Availability

The analyzed data sets generated during the present study are available from the corresponding authors on reasonable request.

## List of Abbreviations

ASRM: American Society for Reproductive Medicine (ASRM)
AMH: Anti-Mullerian hormone
ART: Assisted reproductive technology
BMI: Body mass index
COS: Controlled ovarian stimulation
E_2_: Estradiol
FSH: Follicle-stimulating hormone
GV: Germinal vesicle
GH: Growth hormone
hCG: Human chorionic gonadotropin
ICSI: Intracytoplasmic sperm injection
IVF: In vitro fertilization
LH: Luteinizing hormone
MI: Metaphase I
MPN: Multiple pronuclear
NGS: Next-generation sequencing
PCR: Polymerase chain reaction
PGS: Preimplantation Genetic Screening
PGT-A: Preimplantation genetic testing for aneuploidies
PGT-M: Pre-implantation genetic testing for monogenic/ single gene defects
PRL: Prolactin
SNP: Single-nucleotide polymorphism
T: Testosterone
